# Identifying the Critical Window and Adiposity Trap in Spanish Children: Longitudinal Evolution and Transition Probabilities of BMI Z-Scores from a Clinical Registry (2021–2026)

**DOI:** 10.3390/nu18152455

**Published:** 2026-07-27

**Authors:** Juan P. González-Rivas, María Rodrigo-García, Amparo Rodríguez Sánchez, Álvaro Díaz Conradi, María Zelmira Bosch

**Affiliations:** 1 University Center for Health Sciences–HM Hospitals (CUHMED), Camilo José Cela University, 28660 Madrid, Spain; 2 HM Hospitales Health Research Institute, 28660 Madrid, Spain; mariarodrigog@hmhospitales.com; 3 Department of Epidemiology and Public Health, Harvard TH Chan School of Public Health, Boston, MA 02115, USA; 4 Hospital Universitario HM Montepríncipe, HM Hospitales, 28660 Boadilla del Monte, Spain; amparorodriguezsanchez@yahoo.es; 5 Hospital Universitario de Mataró, Consorci Sanitari del Maresme, 08304 Mataró, Spain; adiaz.conradi@gmail.com; 6 Hospital HM Nens, HM Hospitales, 08009 Barcelona, Spain; mzbosch@ghm.hmhospitales.com

**Keywords:** pediatric obesity, real-world data, Spain

## Abstract

**Background**: The prevalence of pediatric obesity in Spain is among the highest in Europe. Although national surveillance reports a 4.5% decrease in excess weight, these primarily cross-sectional data may underrepresent the metabolic complexity observed in clinical settings. We aim to characterize the evolution of abnormal adiposity and to evaluate the transition probabilities between body mass index (BMI) categories in a multi-regional Spanish cohort. **Methods**: We analyzed Real-World Data (RWD) from 37,465 children and adolescents (ages 0–18) treated at HM Hospitales “Observatorio de la Obesidad Infantil,” between 2021 and 2026. Longitudinal BMI z-score trajectories were evaluated using continuous-time Markov chain models to determine transition probabilities between weight categories and identify developmental windows of adiposity plasticity. **Results**: At baseline, 21.6% of the cohort presented overweight/obesity, with a higher frequency of overweight in females than males (13.0% and 11.2%, respectively; *p* < 0.01) and obesity similar between sexes (~10%). Markov modeling identified a “critical window” in children aged 2–5, who demonstrated a 75% probability of reverting to a normal weight. Conversely, children aged 6–11 entered an “adiposity trap,” characterized by a 60.0% obesity persistence rate and a 23.2% risk of progressing from overweight to obesity. A sharp initial drop in z-scores was observed immediately following the baseline clinical assessment across all groups. **Conclusions**: Our study reveals a significant window of opportunity for abnormal adiposity improvement between the ages of two and five, which contrasts with the adiposity inertia (diminished category fluidity) observed after age five.

## 1. Introduction

Abnormal adiposity in children and adolescents is one of the most pressing public health challenges of the 21st century, with prevalence rates rising dramatically over the past four decades in both high- and low- to middle-income countries [1]. In 2022, more than 390 million children and adolescents aged 5 to 19 were overweight or obese worldwide [1]. The presence of abnormal adiposity at an early age is associated with a wide range of metabolic, hepatic, mechanical, and psychological complications. Consistent evidence shows that insulin resistance, dyslipidemia, and hypertension increasingly appear in affected youth, heightening lifetime risk for type 2 diabetes and cardiovascular disease [2]. In Spanish children, the OBESTIGMA study reported the psychological impact, such as low self-esteem, stigma, and discrimination related to obesity [3]. The ALADINO 2023 study reported a prevalence of 36.1% for excess weight (overweight and obesity) among Spanish schoolchildren, reflecting a 4.5% reduction since 2019 [4]. Despite this decline, Spain remains among the European Union countries with the highest prevalence of pediatric adiposity, with obesity rates remaining particularly persistent in low-income households [5].

While these national surveillance programs provide essential population-level snapshots, significant knowledge gaps persist, limiting clinical and policy responses. First, current data primarily derive from cross-sectional designs, which offer a static view of prevalence, but fail to capture the longitudinal “inertia” or “fluidity” of weight status as children age [6]. There is a lack of mathematical understanding of how and when children transition between BMI categories—data that is essential for identifying the biological and behavioral “traps” that prevent weight normalization [7]. Second, there is a marked scarcity of data from secondary and tertiary hospital settings. Most surveillance occurs in schools, yet the “clinical filter” of hospital care often reveals a population with higher metabolic complexity and a more acute need for multidisciplinary intervention. Electronic Health Records (EHRs) offer a vast source of real-world data (RWD) that remains underutilized within the Spanish pediatric context, particularly for modeling dynamic health state transitions.

Consequently, this study aims to address these gaps by utilizing RWD from the HM Hospitales “Observatorio de la Obesidad Infantil” (2021–2026). Our primary objectives are: (1) to describe the frequency and temporal trends of abnormal adiposity across a multi-regional clinical cohort in Spain; and (2) to apply Markov chain modeling to evaluate the probability of transitioning between body mass index (BMI) categories over time, thereby identifying the specific developmental stages—or “critical windows”—where adiposity remains most plastic versus where it becomes entrenched.

## 2. Methods

### 2.1. Study Design

This is an observational cohort based on the analysis of RWD extracted from the EHRs of pediatric patients treated at HM Hospitales Group centers. The study is part of the Childhood Obesity Observatory, a continuous monitoring initiative promoted by the HM Hospitals Research Foundation (FiHM).

### 2.2. Data Source and Study Population

Data were extracted from the EMR of pediatrics, pediatric endocrinology, and cardiology services across the HM Hospitals Group, spanning the regions of Catalonia, Galicia, Castile and León, and Castile-La Mancha (January 2021 to March 2026). All patients aged 0 to 18 years with at least one record of anthropometric measurements were included.

Duplicate records from the same visit, as well as visits within a span of less than 4 weeks, were consolidated by retaining the most recent value per patient. Temporal trend analysis utilized comparable annual cohorts. A complete-case approach was implemented: individuals missing crucial baseline clinical variables (age, sex, height, or weight) or missing a valid longitudinal date tracker, were excluded from the final analysis. Patients with only a first visit were excluded from the longitudinal analysis.

### 2.3. Anthropometric Variables and Indicators

Weight was measured with a calibrated scale at each center. Height was measured with a stadiometer. BMI was calculated as weight/height^2^ (kg/m^2^). BMI z-score by age and sex was calculated using local tables (specifically, the Spanish pediatric growth references) as a reference: (*BMI-Reference BMI Mean*)/*Reference BMI Standard Deviation* [8]. Weight categories were defined by z-score value as underweight (BMI z-score < −2), Normal weight (z-score ≥ −2 and z-score ≤ 1), overweight (z-score > 1 and z-score ≤ 2), and obesity (z-score > 2).

### 2.4. Ethical Considerations

The study was conducted in accordance with the principles of the Declaration of Helsinki, Spanish data protection legislation (Organic Law 3/2018, LOPDGDD), and the European Union’s General Data Protection Regulation (GDPR 2016/679). Data were anonymized before analysis.

The study protocol was approved by the HM Hospitals Research Ethics Committee in October 2023 (Code 23.10.2241-GHM). Due to the retrospective nature of the study and the use of fully anonymized data, the requirement for informed consent was waived by the Ethics Committee.

### 2.5. Statistical Analysis

Statistical analyses were performed using R version 4.5.3 (R Foundation for Statistical Computing, Vienna, Austria) and the RStudio IDE version 2025.05 (Posit, PBC). Initial descriptive statistics, including means, medians, standard deviations, 95% confidence intervals, and frequency distributions, were generated for all variables, stratified by age, sex, and autonomous community. Prevalence analysis involved estimating the point prevalence of overweight and obesity for each year of the study period. For subgroup comparisons, we established age groups based on standard pediatric developmental milestones and the Spanish educational framework: infants/toddlers (0–24 months), preschoolers (2–5 years), school-age children (6–11 years), and adolescents (12–18 years); sex; and region—we used Chi-square (χ^2^) tests or Fisher’s exact test for categorical variables. Continuous variables were compared using ANOVA for parametric data or Kruskal–Wallis/Mann–Whitney-Wilcoxon tests for non-parametric data, with corrections applied for multiple comparisons.

To analyze the longitudinal evolution of weight status, we utilized continuous-time Markov chain models. These models define the transition space using the established BMI categories, allowing us to evaluate the probability of movement between states over time. We constructed a homogeneous discrete-time Markov chain from longitudinal observations, and the continuous-time models were fitted using an intensity-based framework, with time measured as the continuous interval in years between recorded anthropometric measures. Continuous-time Markov modeling was selected over standard longitudinal techniques (e.g., mixed models) because our primary objective was to quantify the directional probability of moving between distinct clinical health states, rather than to model continuous mean trajectories. Furthermore, Markov frameworks naturally handle highly irregular, unequally spaced real-world clinical follow-up intervals. The continuous-time transition intensity matrix was estimated via Maximum Likelihood Estimation (MLE) using the BFGS optimization algorithm. Interval censoring was handled structurally by computing continuous hazards from transition intensities.

A complete-case approach was implemented, requiring individuals to have at least two recorded visits to model transitions over time. Single-visit patients were excluded from the longitudinal Markov analysis. To evaluate potential selection and attrition biases, a comprehensive baseline comparison was executed between single-visit and multi-visit patients, revealing minor but statistically significant differences in age and baseline obesity distribution (Supplementary Table S1).

The discrete-time chain served as an exploratory layout, while the continuous-time framework yielded the definitive results. The probabilities represent 1-year transitions, calculated from the continuous-time transition intensity matrix. A 1-year time horizon (*t* = 1) was selected for matrix projection to optimize clinical interpretability, aligning directly with standard annual pediatric development visits and Spanish public health surveillance frequencies. Models were stratified by key developmental periods defined strictly by the patient’s age at baseline (2–5 years, 6–11 years, and 12–18 years) to estimate distinct transition probabilities for each cohort. Because stratification was based on baseline age, subsequent transition observations throughout the entire follow-up period contributed to the patient’s baseline-assigned cohort, even if the child chronologically aged into a subsequent developmental period during follow-up. Thus, the transition probabilities represent the long-term longitudinal trajectories of cohorts defined by their entry age. Infants aged 0–24 months were excluded from this specific analysis due to high age-related variability in body weight.

The terms “plasticity” and “trap” are operationally defined based on BMI z-score fluidity and persistence thresholds, respectively. These concepts represent purely statistical constructs derived from longitudinal transition probabilities, rather than direct indicators of proven cellular biological mechanisms or clinical trial intervention effects.

## 3. Results

**Figure 1:**
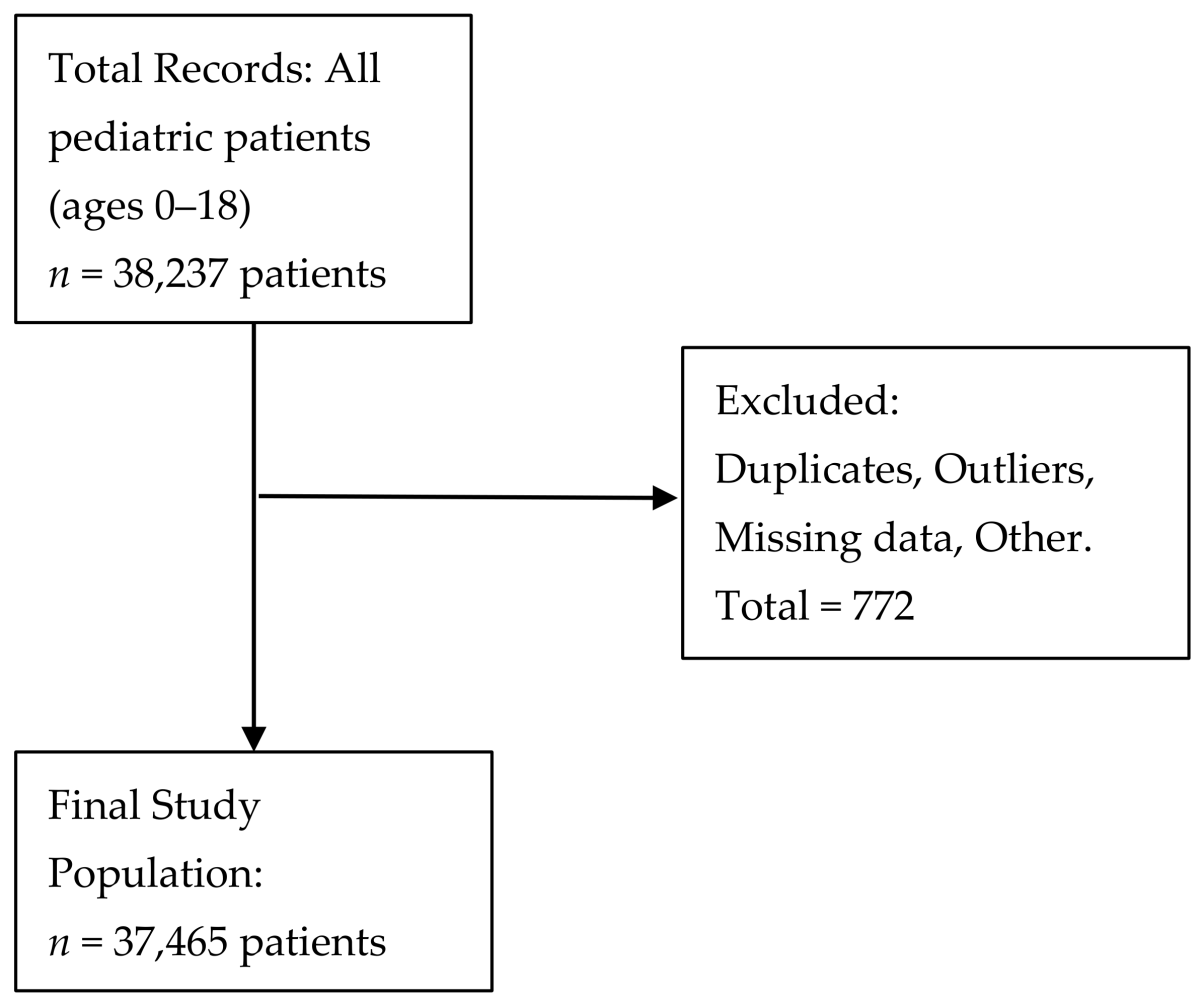
Flowchart of participants included.

The analysis included a total of 37,465 participants from an initial pool of 38,237 individuals, following the removal of duplicate records, outliers, and incomplete datasets (Figure 1). The demographic composition was 50.8% male, with an average age of 6.22 ± 4.83 years across the entire group. The population contributed 102,228 clinical visits, averaging 2.73 ± 2.62 assessments per patient (Table 1). The mean number of follow-up visits was statistically slightly higher in those with underweight and lower with obesity: Underweight 2.97 ± 2.73, Normal weight 2.72 ± 2.62, Overweight 2.80 ± 2.70, and Obesity 2.58 ± 2.46; *p* < 0.001. Most children fell within the 2–5 or 6–11-year age groups (35.7% and 34.7%, respectively). Notably, females had an elevated mean BMI z-score (0.075 ± 1.48) and higher mean BMI percentiles (48.9 ± 33.5) than males (0.034 ± 1.48 and 47.0 ± 33.3, respectively; *p* < 0.001).

### 3.1. Frequency of Abnormal Adiposity at Baseline

**Figure 2:**
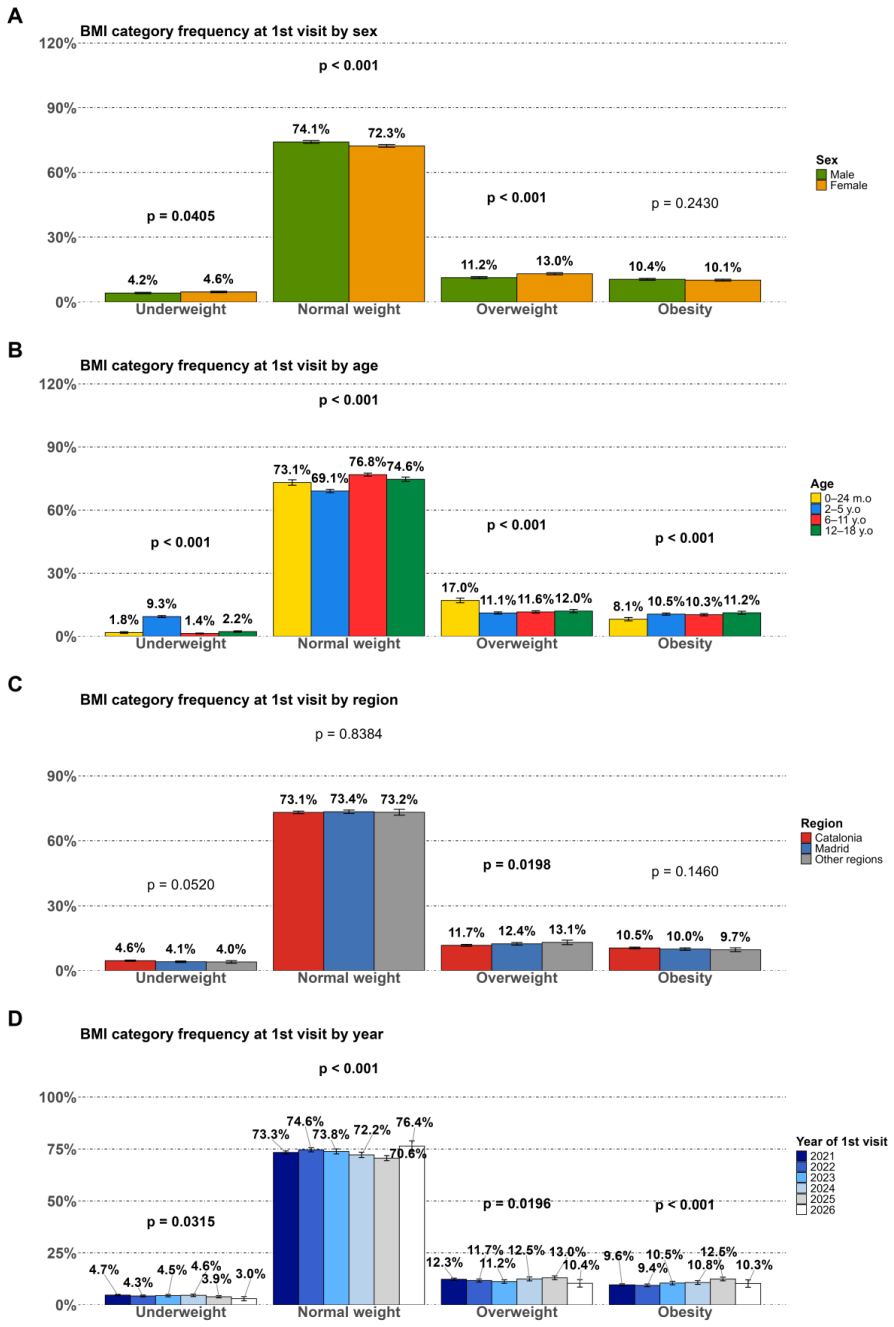
Frequency of abnormal adiposity by sex (**A**), age (**B**), region (**C**), and year of the first visit (**D**). Statistical differences were calculated using Chi-square, with the following *p* values: (**A**)—*p* <0.001; (**B**)—*p* <0.001; (**C**)—*p* = 0.013; (**D**)—*p* < 0.001.

At baseline, the combined frequency of overweight (12.1%) and obesity (10.3%) was 22.4%. The frequency of overweight was higher in females than males (13.0% vs. 11.2%; *p* < 0.001), whereas obesity was similar between sexes (*p* = 0.243) (Figure 2A). By age group, the 2–5-year group had the highest frequency of underweight (9.3%). The frequency of overweight/obesity was different by age group (*p* < 0.001), with those between 0 and 24 months having the highest frequency of overweight (17.0%) but the lowest frequency of obesity (8.1%) (Figure 2B). The highest frequency of obesity was in the age group 12 to 18 years old (11.2%, *p* < 0.001). The Catalonia region had the lowest frequency of overweight (11.7%, *p* = 0.020), and the rest of the categories were similar among regions (Figure 2C). The frequency of overweight and obesity was higher in the years 2024 and 2025, but reduced again in the year 2026 (*p* < 0.05; Figure 2D).

### 3.2. Changes in BMI z-Score over Time by Baseline Weight Category

**Figure 3:**
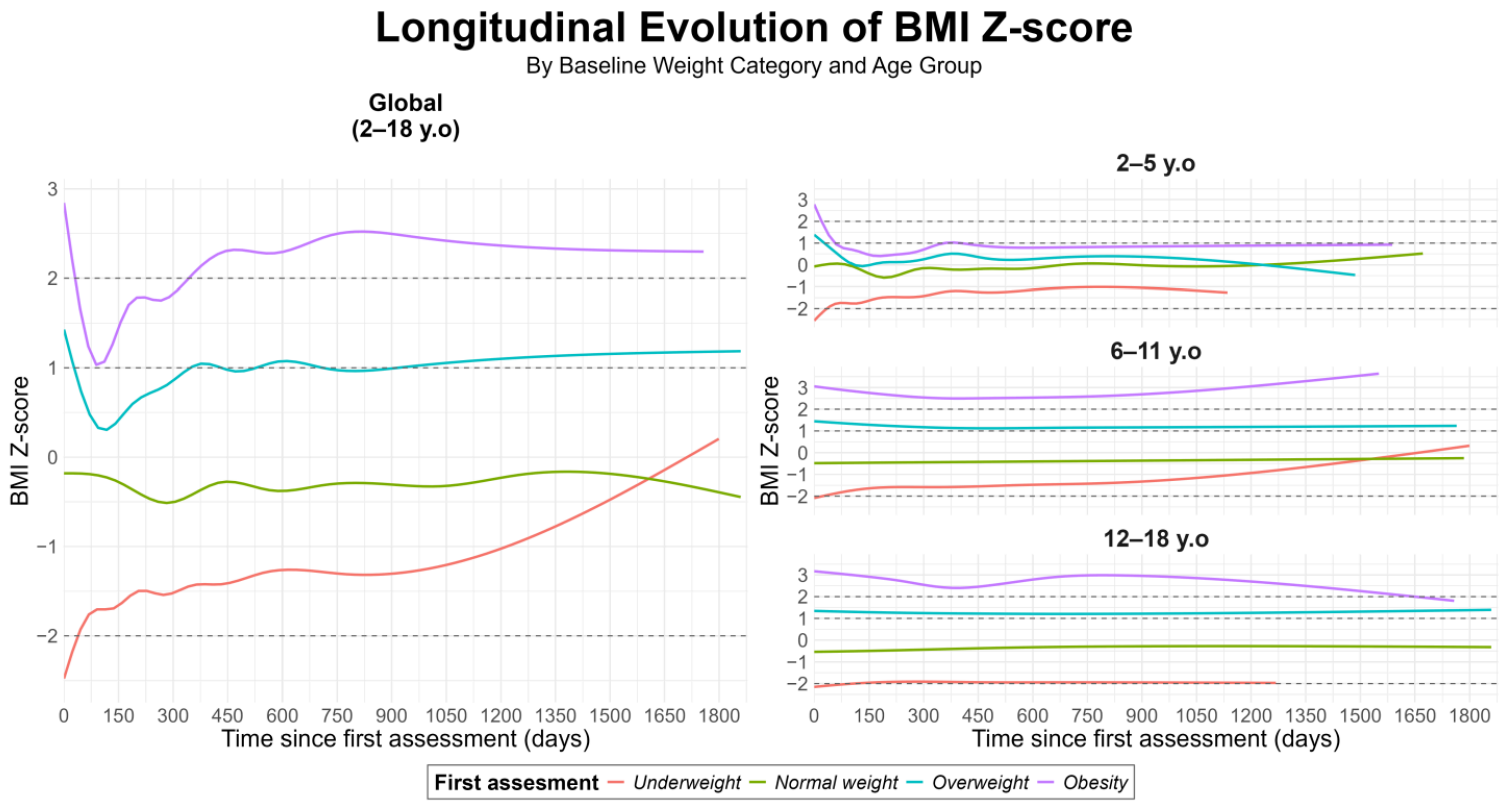
Longitudinal Evolution of BMI Z-scores by Baseline Weight Category and Age Group. The figure displays the temporal changes in BMI Z-scores over a follow-up period of 1800 days (approximately 5 years). The left panel illustrates the trajectories for the aggregate cohort (ages 2–18), while the right panels show age-stratified data for the 2–5, 6–11, and 12–18-year-old groups. Individual lines represent the mean BMI Z-score trajectories based on the patient’s weight status at the initial assessment: Underweight (red), Normal weight (green), Overweight (cyan), and Obesity (purple). Horizontal dashed lines indicate standard Z-score reference thresholds at −2, 0, 1, and 2. The x-axis represents the time in days since the first assessment, and the y-axis represents the BMI Z-score.

The aggregate data for the cohort aged 2–18 years revealed distinct longitudinal trajectories based on the initial assessment nutritional category (Figure 3). Specifically, the underweight group began with the lowest z-scores, of approximately −2.5, and demonstrated a consistent, significant upward trend, approaching a normal z-score above 0 by 1800 days. In contrast, the normal weight group remained the most stable, maintaining a z-score slightly below 0 throughout the five years. Patients categorized as overweight exhibited an initial sharp decline in z-score within the first 150 days, followed by fluctuations and eventual stabilization around 1.1. Similarly, those with obesity experienced an initial rapid decrease in z-score, followed by a period of high variability between 300 and 900 days before stabilizing around 2.3.

When stratified by age, these trends varied significantly across developmental stages (Figure 3). The 2–5-year-old group exhibited a marked regression toward the mean; the obesity group’s z-score dropped from nearly 3.0 to approximately 1.0, while the underweight group showed a gradual increase toward –1.0. Conversely, the 6–11-year-old cohort displayed highly divergent trajectories; while the underweight group showed steady improvement toward a positive z-score, the obesity group followed an upward trend after an initial dip, ending with a z-score of approximately 3.5, which was higher than at baseline. Finally, the 12–18-year-old group exhibited the highest stability for the underweight, normal, and overweight categories, though, notably, the obesity group in this bracket showed a consistent downward trend over time, with z-scores decreasing from 3.2 to approximately 1.8.

### 3.3. Markov Chain Transition Analysis

The Markov chain analysis revealed age-dependent patterns of weight status stability and regression (Table 2). In the 2–5-year-old cohort, transition probabilities were remarkably homogeneous regardless of the initial weight status; all groups showed a high probability—ranging from 75.5% to 76.2%—of transitioning to or maintaining a normal weight by the final assessment. Notably, for this youngest group, the probability of remaining in the obesity category was only 6.0%, suggesting that weight status at this age is highly dynamic and independent of baseline categorization. The probabilities of transitioning to normal weight among regions did not show a statistical difference (Supplementary Table S2). Due to the low baseline prevalence of underweight, regional stratification yielded very small sample sizes and highly unstable estimates (evidenced by extremely wide 95% CIs) in the supplementary models, which must be interpreted with caution.

In contrast, the 6–11-year-old group exhibited significantly higher category-specific persistence and reduced fluidity. Among those with obesity, the probability of persistence was 60.0%. For those starting as overweight, 39.2% returned to a normal weight, while 23.2% progressed to obesity. The 12–18-year-old cohort demonstrated similar stabilization patterns, with a normal weight maintenance probability of 88.5% and an obesity persistence of 58.7%. However, this adolescent group showed a slightly higher probability of reverting from overweight to normal weight (46.6%) compared to the school-aged cohort.

## 4. Discussion

The “Observatorio de la Obesidad Infantil” cohort highlights the significant clinical burden of abnormal adiposity, with baseline excess weight prevalence exceeding 20%. Longitudinal analysis revealed an “initial drop” effect, with BMI z-scores for overweight and obese patients declining sharply following the baseline clinical assessment, suggesting a robust response to initial intervention; however, this sharp initial z-score decline may be substantially driven by statistical regression to the mean (RTM), where extreme baseline values naturally gravitate toward the population average. The findings of change were supported by Markov chain modeling, which indicates nearly universal metabolic plasticity in the 2–5-year age group, where children have a ~75% probability of transitioning to normal weight regardless of baseline status. This fluidity diminishes significantly in the 6–11-year age bracket, where an “adiposity trap” emerges: obesity persistence is 60.0%, and overweight children face a 23.2% risk of progression to obesity. Although the 12–18-year cohort shows higher reversion rates from overweight to normal weight (46.6%)—possibly attributable to pubertal growth or increased health autonomy—the metabolic entrenchment observed after age five confirms that early childhood is the critical window for effective weight normalization. Importantly, because our Markov models track children based on their baseline age cohort, these findings demonstrate that entering clinical observation during the 2–5-year critical window carries a highly favorable prognostic trajectory that persists even as children transition chronologically into school age.

These results provide an important complement—and in some respects a counterpoint—to national surveillance data. The ALADINO 2023 study [4] reported a modest downward trend, with a 4.5% point reduction in excess weight since 2019; however, our hospital-based frequency of obesity among children aged 6 to 11 years (10.3%) is lower than the 15.9% reported for children aged 6 to 9 years at the national level. Rather than indicating a lower burden, this discrepancy likely reflects a combination of age–structure differences and a “clinical trajectory effect,” whereby children attending specialized care may already be undergoing early behavioral or therapeutic changes that reduce BMI at first measurement, as observed in the sharp initial z-score decline across groups. At the same time, our longitudinal design revealed patterns that are not captured by cross-sectional surveillance. The Markov transition probabilities showed a high persistence of obesity in children aged 6–11 years (60.0%) and a substantial risk of progression from overweight to obesity (23.2%), consistent with an “adiposity trap.” Here, we operationally define “adiposity trap” not as an irreversible biological pathway, but as a statistical threshold where the 1-year probability of remaining obese reaches 60.0%, and the risk of moving from overweight to obesity is substantially elevated (23.2%), creating a pattern of strong diagnostic tracking. Compared with Spanish population-based longitudinal cohorts, particularly the ELOIN study in Madrid [9], which reported that approximately 75–77% of children with obesity remained obese during follow-up from ages 4 to 6 and 4 to 9 years, respectively, our clinical registry demonstrated even stronger persistence of severe weight categories at baseline. These factors contribute to Spain’s high rate of pediatric obesity, maintaining the country’s position among the European nations most affected by this condition [5].

Furthermore, although baseline obesity prevalence in early childhood (2–5 years) was comparable to that observed in older groups (~10.5%), this age group demonstrates markedly greater reversibility, with approximately three-quarters transitioning to normal weight, highlighting a critical window of metabolic plasticity that is not captured in population-based surveys such as PASOS 2022, which are restricted to older children (8–16 years) [10]. Our observation of a closing therapeutic window after age five mirrors international longitudinal data, such as the US elementary school cohorts and the UK Millennium Cohort Study, which consistently demonstrate that early childhood weight status is the strongest statistical predictor of persistent adolescent and adult obesity [10,11]. Crucially, the relationship between the preschool critical window and the school-age adiposity trap is sequentially and causally linked within pediatric development [12]. The critical window between ages two and five represents a finite period of elevated physiological and behavioral plasticity. If targeted clinical or lifestyle counter-measures are not successfully established during this highly malleable phase, the physiological architecture—such as the timing of the adiposity rebound and adipocyte hypertrophy—matures and hardens [12]. Consequently, missing this early window of opportunity directly drives the transition into the adiposity trap, transforming a highly fluid and reversible weight status into an entrenched, chronic clinical trajectory after age five.

The observed increase in adiposity inertia after age five necessitates a fundamental shift in public health strategy toward the preschool years. A critical barrier is the parental perception gap: ALADINO 2023 reports that 89.1% of parents of overweight children and 48.8% of parents of children with obesity misperceive their child’s weight as normal [4]. This normalization of excess weight can delay clinical intervention until the child enters the 6–11-year “adiposity trap,” where the probability of spontaneous regression is halved. Furthermore, these findings must be interpreted through the lens of socio-economic equity; while ALADINO 2023 data show improvements in middle- and high-income families, obesity rates remain stubbornly persistent in low-income households [4]. From a biological perspective, the high plasticity observed in the 2–5 age group coincides with the period preceding the physiological adiposity rebound. Conversely, the entrenchment observed after age five aligns with the adiposity rebound window, during which accelerated adipocyte hypertrophy and shifts in insulin sensitivity make weight normalization physiologically more difficult to sustain [13]. The age-stratified transition probabilities observed carry profound clinical and public health implications. The 75.5–76.2% probability of reverting to normal weight among preschoolers (ages 2–5) demonstrates that early childhood is a period of high metabolic and behavioral malleability. Clinically, this justifies a proactive shift away from a passive ‘wait-and-see’ clinical attitude; pediatricians should utilize this highly responsive window for universal early counseling and family-based lifestyle interventions [14]. Conversely, the 60.0% persistence rate observed in school-aged children (ages 6–11) indicates that once obesity is established past age five, it transitions into a highly stable, chronic clinical state [14]. Public health policy must therefore recognize that standard, low-intensity behavioral advice is highly unlikely to succeed in this ‘adiposity trap’. Consequently, clinical guidelines should reinforce early, intensive, multidisciplinary metabolic management, paired with structural public health policies—such as strict school-environment nutrition standards and screen-time reduction initiatives—to break this entrenchment [15]. Environmental factors exacerbate this: children with obesity are more likely to have screen-based devices in their rooms and spend three or more hours daily on sedentary screen time, a trend that increases with age [16]. To be effective, public health interventions must move beyond simple dietary advice and address these structural inequities [17].

The primary strength of this study lies in its use of robust RWD from a multi-regional hospital network, offering a continuous stream of pediatric growth data that traditional cross-sectional or biennial surveys cannot replicate. With a cohort of over 37,000 patients across diverse Spanish autonomous communities, this research provides high statistical power and a granular longitudinal perspective. Unlike school-based surveys, this clinical cohort allowed for the application of Markov chain modeling, uncovering the specific age-dependent transition probabilities and the “adiposity trap” that characterizes the shift from early to middle childhood. However, several limitations warrant consideration. First, as a hospital-based cohort within a private healthcare group, there is an inherent selection bias. The study population likely skews toward a higher socio-economic status compared to the general Spanish population. Our sample reflects families with access to private medical infrastructure, underrepresenting lower-income demographics. Because national data (e.g., ALADINO 2023 [4]) indicate that pediatric obesity is inversely tied to income, our cohort likely overestimates spontaneous reversion rates and underestimates long-term persistence, masking the full extent of the adiposity trap in more vulnerable populations. Second, the reliance on EHR ensures high precision for anthropometric measurements, but limits the availability of uniform data on confounding lifestyle variables—such as caloric intake, specific dietary patterns, or screen time—which are known to significantly influence BMI trajectories in the Spanish context. Third, our registry lacks data on major maternal, early-life, and biological determinants—including parental BMI, birth weight, breastfeeding duration, and pubertal development status (Tanner staging). Consequently, the observed boundaries of our developmental categories may be partly influenced by unmeasured residual confounding, rather than purely isolated chronological windows. Nevertheless, this limitation highlights an important future research direction; subsequent prospective cohorts should combine long-term anthropometric tracking with multiomic assessments of the gut microbiota–metabolites–immune axis—incorporating mechanistic models of intestinal barrier and microenvironmental regulation [18]—to fully elucidate the molecular and cellular pathways that dictate the transition from pediatric tissue plasticity to entrenched adiposity. Fourth, the EHR data lack precise metrics on the specific type, timing, or adherence to clinical interventions, limiting our ability to directly attribute the initial z-score drop to specific therapeutic components. Fifth, differential follow-up frequencies between weight classes pose an inherent selection bias. Our data indicate that children with baseline underweight or normal weight had a slightly higher frequency of follow-up visits compared to those with obesity (2.97 ± 2.73 vs. 2.58 ± 2.46, respectively; *p* < 0.001). This pattern introduces a potential ‘healthy user bias’ (or selective adherence bias), where families with highly motivated health-seeking behaviors or children showing positive progress are more likely to remain active in clinical registries. While continuous-time intensity-based modeling balances irregular time intervals, high obesity persistence could still be partially influenced by more rigid clinical follow-up in severe cases. Sixth, lacking an untreated control group, we cannot disentangle true early therapeutic efficacy from RTM. Seventh, our utilization of local Spanish pediatric growth charts to compute BMI z-scores, while optimizing clinical relevance within the Spanish healthcare system, limits direct comparability with the international literature using World Health Organization standards. Finally, our continuous-time Markov framework structurally required the exclusion of single-visit patients. As demonstrated in Supplementary Table S1, these excluded individuals were slightly older (7.30 vs. 5.33 years) and presented with a marginally higher baseline obesity rate (10.9% vs. 9.8%) than the longitudinal cohort (*p* < 0.001). This indicates an attrition pattern in which older or more severely affected children are less frequently retained for continuous tracking. Consequently, our longitudinal transition estimates should be interpreted with the understanding that they may slightly underestimate the true persistence or severity of abnormal weight categories in the broader, unobserved cross-sectional population.

In conclusion, this longitudinal study utilizing continuous-time Markov chains reveals distinct, age-stratified transition probability patterns in pediatric weight trajectories. Our findings identify the preschool period (ages 2–5) as a highly fluid statistical ‘critical window’ with high probabilities of spontaneous reversion to normal weight, whereas school age (ages 6–11) marks a transition into a highly stable ‘adiposity trap’ characterized by entrenched obesity. While these statistical constructs do not capture underlying biological pathways or direct intervention effects, they carry vital public health implications. Clinical strategies should shift away from a passive ‘wait-and-see’ approach during early childhood, prioritizing proactive, family-centered lifestyle interventions before these metabolic and behavioral trajectories become structurally entrenched.

## Figures and Tables

**Table 1:** Demographic Characteristics at the First Visit.

	Male (*n* = 19,032)	Female (*n* = 18,433)	Overall (*n* = 37,465)	*p*-Value
Total number of visits	52,195	50,033	102,228	
Number of visits/patient	2.74 (2.64)	2.71 (2.61)	2.73 (2.62)	0.416
Age (years)	6.27 (4.90)	6.17 (4.75)	6.22 (4.83)	0.064
Age (months)	74.9 (58.6)	73.6 (56.7)	74.3 (57.7)	0.072
Age (categorized) *n* (%)				<0.001
0–24 months old	2230 (11.7%)	2236 (12.1%)	4466 (11.9%)	0.223
2–5 years old	6876 (36.1%)	6483 (35.2%)	13,359 (35.7%)	0.054
6–11 years old	6299 (33.1%)	6688 (36.3%)	12,987 (34.7%)	<0.001
12–18 years old	3627 (19.1%)	3026 (16.4%)	6653 (17.8%)	<0.001
Height (m)	1.12 (0.364)	1.10 (0.356)	1.11 (0.361)	<0.001
Weight (kg)	24.8 (17.6)	23.9 (16.6)	24.4 (17.1)	0.002
BMI (kg/m^2^)	17.0 (3.06)	16.8 (3.08)	16.9 (3.07)	<0.001
z-score BMI	0.0346 (1.48)	0.0752 (1.48)	0.0546 (1.48)	<0.001
Percentile BMI	47.0 (33.3)	48.9 (33.5)	48.0 (33.4)	<0.001
Weight Category *n* (%)				<0.001
Underweight	798 (4.2%)	854 (4.6%)	1652 (4.4%)	0.405
Normal weight	14,108 (74.1%)	13,324 (72.3%)	27,432 (73.2%)	<0.001
Overweight	2139 (11.2%)	2399 (13.0%)	4538 (12.1%)	<0.001
Obesity	1987 (10.4%)	1856 (10.1%)	3843 (10.3%)	0.243
Region (hospital) *n* (%)				<0.001
Catalonia	11,189 (58.8%)	10,453 (56.7%)	21,642 (57.8%)	<0.001
Madrid	6184 (32.5%)	6277 (34.1%)	12,461 (33.3%)	0.001
Other Regions	1593 (8.4%)	1629 (8.8%)	3222 (8.6%)	0.107
Region (patient) *n* (%)				
Catalonia	11,028 (57.9%)	10,299 (55.9%)	21,327 (56.9%)	<0.001
Madrid	5938 (31.2%)	6047 (32.8%)	11,985 (32.0%)	<0.001
Other Regions	2066 (10.9%)	2087 (11.3%)	4153 (11.1%)	0.155

Abbreviations: BMI—Body Mass Index. The term ‘regions’ refers to the Autonomous Communities of Spain. Continuous data is presented as mean ± SD, and categorical variables as *n* (%).

**Table 2:** Markov Chain Transition Probabilities by Age Group.

Age Group	First Assessment	Last: UW	Last: NW	Last: OW	Last: OB
2–5 years old	UW	0.110 (0.105–0.116)	0.762 (0.756–0.769)	0.075 (0.072–0.079)	0.052 (0.049–0.056)
	Normal weight	0.109 (0.104–0.114)	0.761 (0.755–0.768)	0.076 (0.072–0.079)	0.054 (0.050–0.057)
	Overweight	0.107 (0.102–0.112)	0.759 (0.752–0.765)	0.078 (0.074–0.081)	0.057 (0.053–0.060)
	Obesity	0.105 (0.100–0.110)	0.755 (0.748–0.762)	0.080 (0.076–0.083)	0.060 (0.056–0.064)
6–11 years old	UW	0.242 (0.167–0.323)	0.713 (0.639–0.782)	0.038 (0.032–0.044)	0.007 (0.006–0.009)
	NW	0.007 (0.005–0.009)	0.906 (0.898–0.913)	0.070 (0.064–0.075)	0.018 (0.016–0.021)
	OW	0.002 (0.001–0.003)	0.392 (0.361–0.425)	0.374 (0.346–0.402)	0.232 (0.211–0.255)
	OB	0.000 (0.000–0.001)	0.123 (0.108–0.140)	0.277 (0.253–0.303)	0.600 (0.562–0.631)
12–18 years old	UW	0.164 (0.107–0.236)	0.780 (0.714–0.834)	0.045 (0.038–0.053)	0.010 (0.008–0.013)
	NW	0.024 (0.019–0.029)	0.885 (0.874–0.895)	0.070 (0.062–0.078)	0.022 (0.018–0.026)
	OW	0.009 (0.007–0.011)	0.466 (0.429–0.507)	0.295 (0.265–0.324)	0.231 (0.200–0.263)
	OB	0.002 (0.002–0.003)	0.158 (0.133–0.186)	0.253 (0.221–0.285)	0.587 (0.531–0.637)

Data are presented as transition probabilities and corresponding 95% confidence intervals (95% CI). **Abbreviations:** UW: Underweight; NW: Normal weight; OW: Overweight; OB: Obesity.

## Data Availability

The data that support the findings of this study are available from the corresponding author upon reasonable request.

## Supplementary Materials

The following supporting information can be downloaded at: https://www.mdpi.com/article/10.3390/nu18152455/s1, Table S1: Differences between patients who attended one or more visits; Table S2: Markov Chain Transition Probabilities by Age Group and Regions.

## Abbreviations

ANOVA	Analysis of Variance
BMI	Body Mass Index
CI	Confidence Interval
EHR(s)	Electronic Health Record(s)
EMR	Electronic Medical Record
FiHM	Fundación de Investigación HM Hospitales (HM Hospitals Research Foundation)
GDPR	General Data Protection Regulation
LOPDGDD	Organic Law on Data Protection and Digital Rights Guarantee (Spain)
NW	Normal Weight
OB	Obesity
OW	Overweight
RWD	Real-World Data
SD	Standard Deviation
UW	Underweight
χ^2^ (Chi-square)	Chi-square test
