# Supplementary material for: Identifying the Critical Window and Adiposity Trap in Spanish Children: Longitudinal Evolution and Transition Probabilities of BMI Z-Scores from a Clinical Registry (2021–2026)
Source: Nutrients. 2026 Jul 27;18(15):2455. doi: 10.3390/nu18152455 (PMC13468087; doi:10.3390/nu18152455)
Supplement: Supplementary file 1 [file nutrients-18-02455-s001.zip › nutrients-4406506-supplementary.pdf]

Supplementary Table S1: Differences between patients who attended one or more visits

	1 visit (n=16942)	>2 visits (n=20523)	Total (N=37465)	p-value
Number of visits/patient	1.00 (±0.00)	4.49 (±3.31)	2.91 (±3.00)	<0.001
Sex				0.456
Male	8570 (50.6%)	10462 (51.0%)	19032 (50.8%)	
Female	8372 (49.4%)	10061 (49.0%)	18433 (49.2%)	
Age (years)	7.30 (±4.81)	5.33 (±4.65)	6.22 (±4.83)	<0.001
BMI Z-score	0.0909 (±1.50)	0.0246 (±1.45)	0.0546 (±1.48)	<0.001
BMI (categorized by z-score)				<0.001
Underweight	688 (4.1%)	964 (4.7%)	1652 (4.4%)	0.003
Normal weight	12420 (73.3%)	15012 (73.1%)	27432 (73.2%)	0.734
Overweight	1992 (11.8%)	2546 (12.4%)	4538 (12.1%)	0.058
Obesity	1842 (10.9%)	2001 (9.8%)	3843 (10.3%)	<0.001

Abbreviations: BMI - Body mass index

Supplementary Table S2. Markov Chain Transition Probabilities by Age Group and Regions

Region	Age Group	First Assessment	Last: UW	Last: HW	Last: OW	Last: OB
Catalonia	2-5 years	UW	0.165 (0.153-0.178)	0.682 (0.670-0.693)	0.089 (0.084-0.093)	0.064 (0.059-0.069)
Catalonia	2-5 years	HW	0.147 (0.137-0.155)	0.690 (0.681-0.700)	0.092 (0.087-0.096)	0.072 (0.066-0.077)
Catalonia	2-5 years	OW	0.143 (0.134-0.152)	0.688 (0.678-0.697)	0.092 (0.088-0.097)	0.077 (0.071-0.083)
Catalonia	2-5 years	OB	0.129 (0.121-0.137)	0.674 (0.663-0.684)	0.096 (0.092-0.100)	0.101 (0.091-0.112)
Catalonia	6-11 years	UW	0.115 (0.065-0.184)	0.827 (0.763-0.871)	0.048 (0.041-0.056)	0.010 (0.008-0.012)
Catalonia	6-11 years	HW	0.009 (0.007-0.013)	0.901 (0.891-0.910)	0.070 (0.063-0.078)	0.019 (0.016-0.023)
Catalonia	6-11 years	OW	0.004 (0.003-0.005)	0.450 (0.413-0.487)	0.323 (0.292-0.354)	0.223 (0.195-0.252)
Catalonia	6-11 years	OB	0.001 (0.001-0.001)	0.139 (0.118-0.161)	0.250 (0.219-0.279)	0.611 (0.568-0.657)
Catalonia	12-18 years	UW	0.317 (0.201-0.426)	0.624 (0.525-0.727)	0.050 (0.040-0.062)	0.009 (0.006-0.012)
Catalonia	12-18 years	HW	0.014 (0.009-0.019)	0.858 (0.841-0.873)	0.103 (0.090-0.115)	0.026 (0.021-0.032)
Catalonia	12-18 years	OW	0.004 (0.003-0.007)	0.418 (0.364-0.473)	0.361 (0.312-0.409)	0.216 (0.179-0.260)
Catalonia	12-18 years	OB	0.001 (0.001-0.001)	0.129 (0.102-0.160)	0.263 (0.220-0.307)	0.607 (0.542-0.670)
Madrid	2-5 years	UW	0.096 (0.061-0.797)	0.720 (0.215-0.926)	0.127 (0.002-0.120)	0.057 (0.000-0.094)
Madrid	2-5 years	HW	0.096 (0.047-0.252)	0.720 (0.672-0.935)	0.127 (0.002-0.160)	0.057 (0.000-0.099)
Madrid	2-5 years	OW	0.096 (0.038-0.210)	0.720 (0.633-0.914)	0.127 (0.011-0.162)	0.057 (0.000-0.130)
Madrid	2-5 years	OB	0.096 (0.038-0.197)	0.720 (0.584-0.910)	0.127 (0.016-0.169)	0.057 (0.002-0.140)
Madrid	6-11 years	UW	0.958 (0.227-0.999)	0.041 (0.001-0.737)	0.001 (0.000-0.027)	0.000 (0.000-0.007)
Madrid	6-11 years	HW	0.006 (0.002-0.013)	0.927 (0.911-0.939)	0.049 (0.039-0.060)	0.018 (0.013-0.023)
Madrid	6-11 years	OW	0.002 (0.001-0.004)	0.463 (0.398-0.533)	0.288 (0.237-0.343)	0.247 (0.196-0.302)
Madrid	6-11 years	OB	0.001 (0.000-0.001)	0.205 (0.160-0.262)	0.301 (0.252-0.349)	0.493 (0.409-0.570)
Madrid	12-18 years	UW	0.028 (0.018-0.057)	0.919 (0.892-0.933)	0.043 (0.033-0.055)	0.010 (0.006-0.015)
Madrid	12-18 years	HW	0.022 (0.016-0.031)	0.917 (0.899-0.931)	0.048 (0.037-0.061)	0.013 (0.008-0.020)
Madrid	12-18 years	OW	0.012 (0.009-0.017)	0.575 (0.492-0.655)	0.228 (0.170-0.294)	0.184 (0.129-0.252)
Madrid	12-18 years	OB	0.003 (0.002-0.005)	0.173 (0.115-0.256)	0.201 (0.139-0.269)	0.622 (0.485-0.734)

Other Regions	2-5 years	UW	0.122 (0.114-1.000)	0.738 (0.000-1.000)	0.084 (0.000-1.000)	0.056 (0.000-1.000)
Other Regions	2-5 years	HW	0.118 (0.000-1.000)	0.741 (0.000-1.000)	0.084 (0.000-1.000)	0.057 (0.000-1.000)
Other Regions	2-5 years	OW	0.118 (0.000-1.000)	0.741 (0.000-1.000)	0.084 (0.000-1.000)	0.057 (0.000-1.000)
Other Regions	2-5 years	OB	0.117 (0.000-1.000)	0.742 (0.000-1.000)	0.084 (0.000-1.000)	0.057 (0.051-1.000)
Other Regions	6-11 years	UW	0.270 (0.096-0.487)	0.691 (0.490-0.854)	0.032 (0.020-0.046)	0.006 (0.004-0.010)
Other Regions	6-11 years	HW	0.007 (0.004-0.014)	0.915 (0.894-0.931)	0.062 (0.050-0.077)	0.016 (0.012-0.022)
Other Regions	6-11 years	OW	0.002 (0.001-0.004)	0.433 (0.369-0.503)	0.346 (0.290-0.405)	0.218 (0.173-0.271)
Other Regions	6-11 years	OB	0.001 (0.000-0.001)	0.148 (0.114-0.197)	0.284 (0.232-0.341)	0.568 (0.482-0.645)
Other Regions	12-18 years	UW	0.355 (0.077-0.692)	0.599 (0.287-0.841)	0.036 (0.015-0.063)	0.010 (0.004-0.019)
Other Regions	12-18 years	HW	0.018 (0.009-0.034)	0.875 (0.837-0.903)	0.078 (0.058-0.105)	0.030 (0.020-0.044)
Other Regions	12-18 years	OW	0.006 (0.003-0.012)	0.453 (0.365-0.551)	0.284 (0.219-0.357)	0.256 (0.185-0.336)
Other Regions	12-18 years	OB	0.002 (0.001-0.004)	0.191 (0.124-0.277)	0.283 (0.205-0.359)	0.524 (0.389-0.652)

Abbreviations: UW: Underweight; NW: Normal weight; OW: Overweight; OB: Obesity.

Note on Parameter Instability: Readers are cautioned that due to the exceptionally low baseline frequency of underweight children in the cohort, sub-stratification by region and age group resulted in extremely small cell counts for several transition pathways (particularly the 2–5 age bracket in Madrid and 'Other Regions'). This sample size limitation is reflected in the extremely wide 95% confidence intervals and boundary estimates (intervals reaching infinity) for these specific rows. These regional subgroup estimates are highly unstable and should be interpreted purely as exploratory.
